# Integrating Strategy of Network Pharmacology, Molecular Dynamics Simulation, and Experimental Verification to Investigate the Potential Mechanism of *Gastrodia elata* Against Alcoholic Liver Injury

**DOI:** 10.3390/foods14122008

**Published:** 2025-06-06

**Authors:** Peiyuan Sun, Ruohan Zhang, Xuanyou Li, Dengwang Yang, Shunfeng Ji, Lei Peng, Jun Sheng, Jing Wang

**Affiliations:** 1 Key Laboratory of Development and Utilization of Food and Medicinal Resources, Ministry of Education, Yunnan Agricultural University, Kunming 650201, China; sunxingjia8888@126.com (P.S.); 15215003760@163.com (R.Z.); lxuanyoug@163.com (X.L.); 2 College of Science, Yunnan Agricultural University, Kunming 650201, China; 18146259573@163.com (D.Y.); m17787251480@163.com (S.J.); 3 College of Food Science and Technology, Yunnan Agricultural University, Kunming 650201, China; 2015042@ynau.edu.cn

**Keywords:** *Gastrodia elata*, alcoholic liver injury, network pharmacology, molecular dynamics simulation, cell experiment, molecular mechanism

## Abstract

As one of the medicinal and edible resources, *Gastrodia elata* (GE) is considered to hold potential in alleviating alcoholic liver injury, yet its mechanism needs further elucidation. To explore the molecular mechanisms of GE against alcoholic liver injury, network pharmacology, molecular docking, molecular dynamics simulations, and cell experiments were employed. Thirty-two active components of GE may exert efficacy against alcohol-induced liver injury via regulating 207 targets. Among them, the main functional components might be 4-hydroxybenzyl methyl ether, 4-ethoxytolyl-4′-hydroxybenzyl ether, pseudolaric acid B, palmitic acid, and myricetin. Analyses of Gene Ontology (GO) enrichment and Kyoto Encyclopedia of Genes and Genomes (KEGG) pathway enrichment showed that a total of 322 GO items and 154 KEGG pathways are related to the effects of GE against alcoholic liver injury. The results of molecular docking show that the main active components of GE might interact with the key target proteins of GAPDH, PPARG, EGFR, STAT3, and AKT1. Molecular dynamics simulation further determined that pseudolaric acid B, as the core component, stably binds to these key target proteins. Cell experiments demonstrate that pseudolaric acid B exhibits a protective effect on ethanol-induced HepG2 cell injury by down-regulating the protein expression levels of GAPDH, STAT3, PPARG, and EGFR. Furthermore, the agent also suppresses IL-6 and inhibits the abnormal absorption of total cholesterol in HepG2 cells. Our findings suggest the efficacy and mechanism of GE in combating alcoholic liver injury and lay the groundwork for the precise development and utilization of GE.

## 1. Introduction

The liver is the primary organ for ethanol metabolism in the human body. Ethanol is metabolized to acetaldehyde by alcohol dehydrogenase in hepatocytes, which subsequently impairs protein function and repair, leading to alcoholic liver injury characterized by oxidative stress, lipid accumulation, and inflammation [1]. It has been reported that the incidence of alcohol-induced liver injury is on the rise year by year. Moreover, with the increasing rate of alcohol abuse among young people, the age of patients with alcoholic liver disease (ALD) is also trending younger [2]. In China, over 20% of the population is affected by liver diseases, with alcoholic liver injury accounting for the majority of cases. Moreover, alcoholic liver injury is responsible for 50% of global cirrhosis-related deaths, posing a significant threat to public health [3,4]. Current treatments for ALD, including surgical interventions and pharmacological therapies, are often associated with substantial adverse effects and high recurrence rates [5]. Therefore, developing safer, low-toxicity hepatoprotective products from natural products is a crucial approach for the prevention and treatment of alcoholic liver injury.

China is rich in medicinal and edible plant resources. These resources, owing to their functional components, exert a positive impact on human health [6,7]. *Gastrodia elata* Bl. (GE), a traditional Chinese medicine with a long history, is officially listed in the list of food and medicine homologies in China and holds great potential for food development [8]. Therefore, enhancing the investigation of the bioactivities and mechanisms of the functional components of GE is of great significance for its development and utilization in the functional food sector. In recent years, it has been reported that GE holds significant potential for the prevention and treatment of liver disease [9,10]. However, the bioactive components and underlying molecular mechanisms of GE against alcoholic liver injury need further investigation. This limitation restricts the precise development and utilization of GE for alleviating alcoholic liver injury. To investigate the active components and mechanisms underlying anti-alcoholic liver injury efficacy of GE, the integration of network pharmacology, molecular docking, and molecular dynamics simulation provides a practical and effective approach for researchers.

As a subset of systems biology, network pharmacology offers a comprehensive framework for unraveling the pivotal molecular pathways that underpin the therapeutic efficacy of traditional Chinese medicine and its formulations [6,11]. Molecular docking, a method of computer-aided drug design, simulates the binding models between ligands and receptors to predict their molecular recognition mechanisms [12]. This technique enables rapid identification of lead compounds during drug screening and provides quantitative assessments of binding affinities between small molecules and target proteins, thereby underpinning the theoretical foundations of novel drug development. Additionally, molecular dynamics simulation has garnered significant attention as a powerful and effective approach recently. It elucidates the temporal dynamics of interactions between macromolecules and small molecules, offering critical insights into the binding processes of lead compounds with their targets and corroborating the outcomes of molecular docking studies [13,14]. Therefore, in this study, we investigate the potential effects and molecular mechanisms of GE in ameliorating alcoholic liver injury based on an integration of network pharmacology, molecular docking, molecular dynamics simulation, and cell experiments, aiming to provide scientific evidence for its application in functional foods and the development of novel treatments for alcoholic liver injury.

## 2. Materials and Methods

### 2.1. Materials

HepG2 cells were provided by the Kunming Cell Bank of the Chinese Academy of Sciences (Kunming, China). Pseudolaric Acid B (B21265) was purchased from Yuanye Biotechnology Co., Ltd. (Shanghai, China). Dulbecco’s Minimal Essential medium was purchased from Dalian Meilun Biotechnology Co., Ltd. (Dalian, China). Fetal bovine serum (FBS) was purchased from Grand Island Biological Co., Ltd. (Grand Island, NE, USA). Penicillin/streptomycin (P/S), trypsin-EDTA solution, RIPA lysis buffer, 30% acrylamide gel solution, and methyl thiazolyl tetrazolium (MTT) were purchased from Solarbio (Beijing, China). EGFR, STAT3, GAPDH, and IL-6 antibodies were purchased from ABclonal (Wuhan, China). β-Actin antibody was purchased from BioWorld (Bloomington, MN, USA). PPARG antibody was purchased from ABmart (Shanghai, China). A total cholesterol assay kit was purchased from Nanjing Jiancheng Bioengineering Institute (Nanjing, China).

### 2.2. Determination of Bioactive Components and Gene Targets Related to Alcoholic Liver Injury

The potential bioactive components and targets of GE were obtained from the TCMID database (https://www.bidd.group/TCMID/b.org/, accessed on 9 May 2024), and then the PubChem database (https://pubchem.ncbi.nlm.nih.gov/, accessed on 12 May 2024) was utilized to obtain the SDF structure of the above active components. The Uniprot database (http://www.uniprot.org/uniprot/, accessed on 13 May 2024) was utilized to predict the gene targets of GE components targets with a rating of >0. Potential targets of alcoholic liver injury were obtained through the OMIM database (https://omim.org/, accessed on 14 May 2024) and GeneCards disease database (https://www.genecards.org/, accessed on 14 May 2024). Venny 2.1.0 (https://bioinfogp.cnb.csic.es/tools/venny/, accessed on 16 May 2024) was used to analyze the intersected targets of GE and alcoholic liver injury.

### 2.3. Analysis of Protein–Protein Interaction (PPI) Network

The potential targets of GE for ameliorating alcoholic liver injury were imported into the STRING analysis platform (https://string-db.org/, accessed on 18 May 2024), and then the species was set as Homo sapiens and the minimum medium confidence was set to 0.40. After obtaining PPI network relationship data, Cytoscape 3.10.0 software was used to construct the PPI network.

### 2.4. GO Enrichment and KEGG Pathway Enrichment Analysis

The potential targets of GE for treating alcoholic liver injury were imported into the Metascape database, and “Homo Sapiens” was selected to input and analyze as the species. The minimum overlap value was set as 3, and the *p* value cutoff was set as 0.01, and the minimum enrichment value was set as 1.5. Finally, GO and KEGG enrichment analyses were conducted to analyze potential targets.

### 2.5. Molecular Docking Simulation

The target proteins and bioactive components with high degree values in PPI network were selected as receptors and ligands, respectively, for subsequent molecular docking studies. The crystal structures of these key target proteins were sourced from the Protein Data Bank (PDB) database (http://www.rcsb.org, accessed on 28 May 2024) and employed as the docking receptors. AutoDock Tools 1.56 was utilized for grid generation and docking simulations. The centers of grid used for the docking search were determined according to the literature [6,13,15,16,17]. The docking parameters were mostly set to default, with specific adjustments made to increase the number of genetic algorithm (GA) runs to 20 and set the maximum number of evaluations (medium) to 5,000,000. Ultimately, the docking pose with the lowest binding energy was chosen as the optimal configuration. The receptor–ligand interactions were further analyzed and visualized using DiscoveryStudio 2024 and PyMol 1.5 software.

### 2.6. Molecular Dynamics Simulation

Utilizing the optimal docking model obtained from molecular docking, a molecular dynamics (MD) simulation was performed using the Gromacs-2020.6 software suite. The AMBER99SB force field and the simple point charge (SPC) water model were employed to characterize the receptor proteins. A cubic simulation box was established to encompass the receptor proteins, and counterions (Na^+^ or Cl^−^) were added to neutralize the net charge of the system. For structural relaxation, energy minimization was performed with a combination of steepest descent and conjugate gradient algorithms, each set to 5000 steps. Subsequently, the minimized system was gradually heated within a normal volume temperature (NVT) ensemble, with the temperature rising from 0 to 300 K over a period of 1 ns. Following this, the system underwent density equilibration under a normal pressure temperature (NPT) ensemble, maintained at 300 K for 1 ns. Ultimately, the MD simulation was run for 100 ns to investigate the dynamic binding characteristics of the receptor–ligand complexes.

### 2.7. Cell Culture

HepG2 cells were cultured in DMEM medium supplemented with 10% FBS and 1% P/S. The cells were maintained in a humidified incubator at 37 °C with 5% CO_2_, and the culture medium was replaced every two days.

### 2.8. MTT Assay

HepG2 cells were seeded into a 96-well plate at a density of 2 × 10^4^ cells per well, and stimulated with different concentrations of ethanol (0, 50, 100, 200, 400, 800 mmol/L) for 24 h in a humidified incubator at 37 °C with 5% CO_2_. After adding 20 μL of MTT solution and incubating for 4 h in the dark, the supernatant was removed and 200 μL of dimethyl sulfoxide (DMSO) was added to each well. Finally, the value of OD in each well was measured at 492 nm using FlexStation 3 microplate reader (Molecular Devices, Shanghai, China).

After the concentration of ethanol was determined, HepG2 cells were treated using 10 and 20 μM of pseudolaric acid B for 24 h and then were stimulated with 400 mmol/L of ethanol for 24 h. The cells were finally used for MTT assay, and the value of OD in each well was measured at 492 nm.

### 2.9. Western Blotting

HepG2 cells were seeded in 60 mm plates at a density of 2 × 10^6^ cells per plate and then treated with 10 μM or 20 μM of pseudolaric acid B for a duration of 24 h in a humidified incubator at 37 °C with 5% CO_2_. After stimulation with 400 mmol/L of ethanol for 24 h, protein extraction was performed using a mixture of RIPA buffer and PMSF (Solarbio, Beijing, China) in a population of 100:1. Equivalent quantities of protein samples were resolved by sodium dodecyl sulfate–polyacrylamide gel electrophoresis (SDS-PAGE) and transferred onto PVDF membranes. The membranes were blocked with 10 mL of 5% non-fat milk in TBS buffer containing 0.1% Tween-20 for 1 h at room temperature. Following this, they were incubated with GAPDH, PPARG, EGFR, STAT3, and IL-6 antibodies at 4 °C overnight, and then incubated with secondary antibodies for 1 h at room temperature. Finally, the membranes were subjected to analysis using the FluorChem E System (Santa Clara, CA, USA).

### 2.10. Detection of Total Cholesterol

HepG2 cells were plated in 60 mm dishes at a density of 2 × 10^6^ cells per dish. The cells were treated with various doses of pseudolaric acid B for 24 h and then treated with 400 mmol/L of ethanol for 24 h. Subsequently, protein extraction was conducted according to step 2.9. Finally, the prepared protein extracts were tested using a total cholesterol assay kit and measured at 500 nm using a FlexStation 3 microplate reader.

### 2.11. Statistical Analysis

SPSS 26.0 and Graphpad Prism 8.0 software were used for statistical analysis. All experiments were performed independently at least thrice. The data is presented as (mean ± SEM), and comparisons between multiple groups were conducted using a one-way analysis of variance (ANOVA) followed by Duncan’s multiple test. Statistical significance was set at *p* < 0.05.

## 3. Results

### 3.1. Construction of Intersection Target Network Between GE and Alcoholic Liver Injury

Firstly, a total of 34 bioactive components of GE were identified in the TCMID database. After eliminating duplicates and components without targets, 31 bioactive components were ultimately selected (Table 1). The Swiss Target Prediction Database was employed to remove redundant targets, resulting in the identification of 494 target genes. Relevant targets associated with alcoholic liver injury were collected from the OMIM and GeneCards disease databases. With a relevance score threshold of ≥10, targets that did not meet this criterion were excluded, yielding 1243 targets related to alcoholic liver injury.

**Figure 1:**
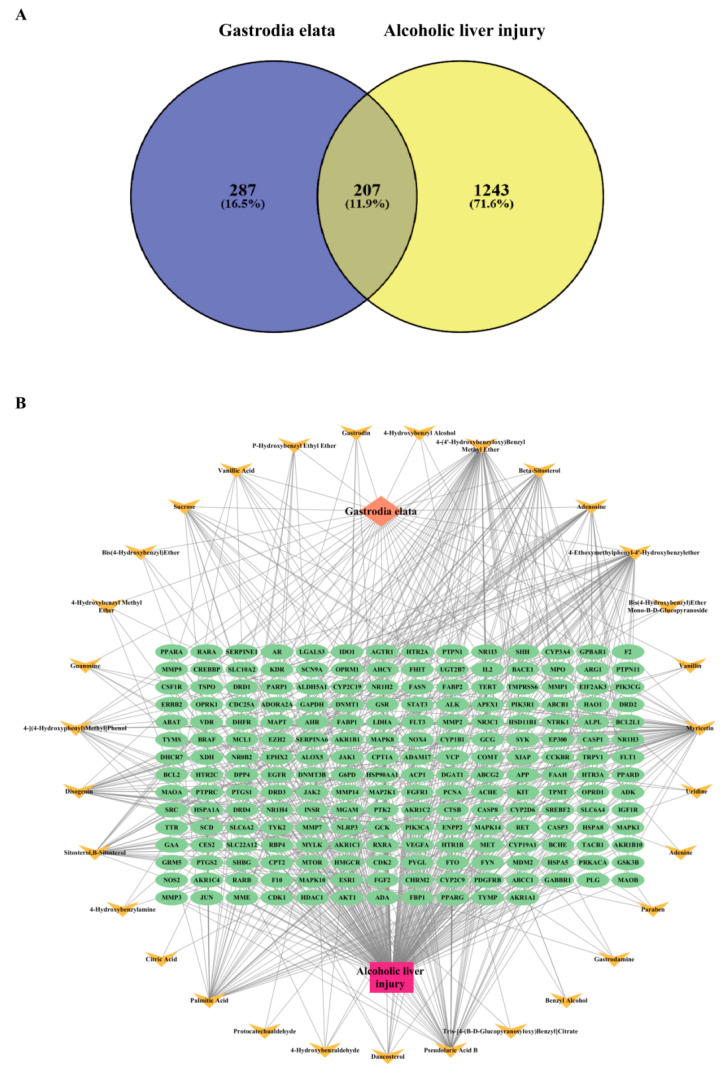
Identification of bioactive components in *Gastrodia elata* for ameliorating alcoholic liver injury. (**A**) The intersection between gene targets of bioactive components derived from GE and gene targets of alcoholic liver injury is depicted by a Venn diagram. (**B**) The “GE-bioactive components-targets-alcoholic liver injury” network graph was analyzed by Cytoscape 3.10.0. The yellow nodes represent the gene targets of GE, while the green nodes represent the gene targets of alcoholic liver injury.

Next, the Venny 2.1 software platform was utilized to obtain the intersecting targets between the active components of GE and alcoholic liver injury, resulting in 207 target genes, as shown in Figure 1A. These intersecting targets represent the potential targets of GE that can act on alcoholic liver injury. Then, we employed Cytoscape 3.10.0 software to construct a “*Gastrodia elata*-bioactive components-targets-alcoholic liver injury” network (Figure 1B), where two distinct colors were used to represent bioactive components and the common targets shared by bioactive components and alcoholic liver injury, respectively. Based on the degree value, the top five components in the network were identified, which are 4-(4′-hydroxybenzyloxy)benzyl methyl ether, 4-ethoxymethylphenyl-4′-hydroxybenzylether, pseudolaric acid B, palmitic acid, and myricetin, respectively.

### 3.2. Analysis of PPI Network of Targets Between Gastrodia elata and Alcoholic Liver Injury

**Figure 2:**
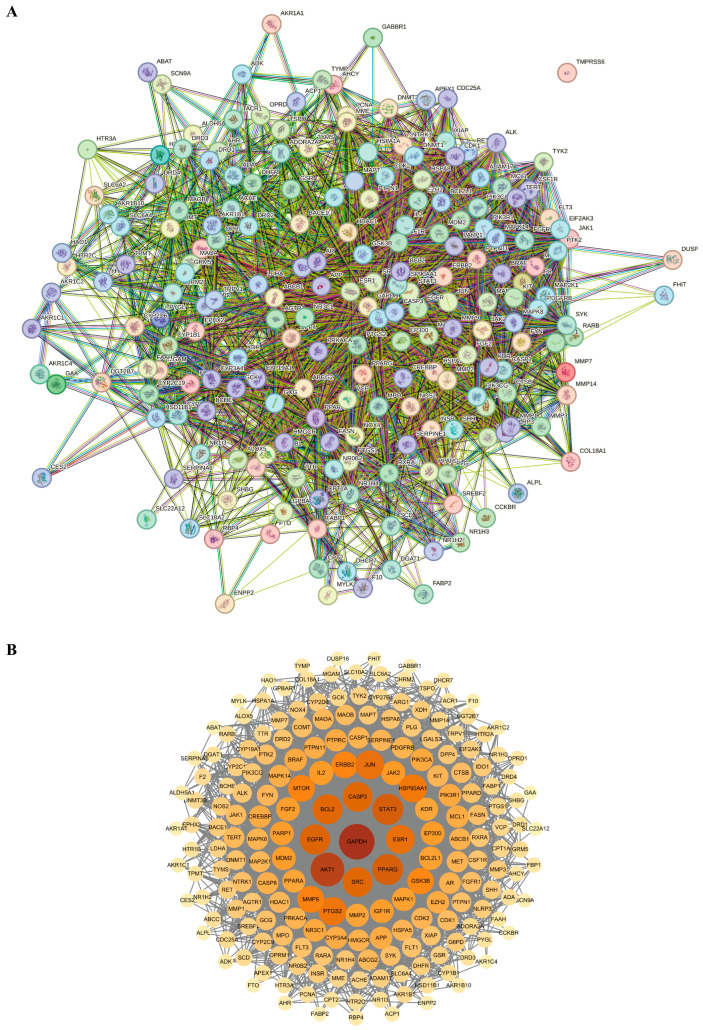
Identification of the key targets of bioactive components derived from GE against alcoholic liver injury. (**A**) The PPI network diagram was constructed using the STRING database. (**B**) The core targets was identified by using Cytoscape software. Nodes indicate targets proteins, while edges represent the connections between target proteins. Larger and darker red nodes represent more important target proteins in the network.

Protein–protein interactions (PPI) refer to the process by which two or more protein molecules form protein complexes through non-covalent bonds. This process is of fundamental importance to the majority of biological functions and processes. Therefore, the information of the intersecting target genes between GE and alcoholic liver injury was subsequently imported into the STRING database for PPI network analysis and then visualized using Cytoscape 3.10.0 software. In this network, the degree values were represented by variations in node size, color, and color intensity. Specifically, nodes with darker colors and larger sizes corresponded to higher degree values, as shown in Figure 2. Based on the degree values, the top five core targets identified were GAPDH, AKT1, STAT3, PPARG, and EGFR.

### 3.3. GO and KEGG Enrichment Analysis of Potential Targets of Gastrodia elata Against Alcoholic Liver Injury

**Figure 3:**
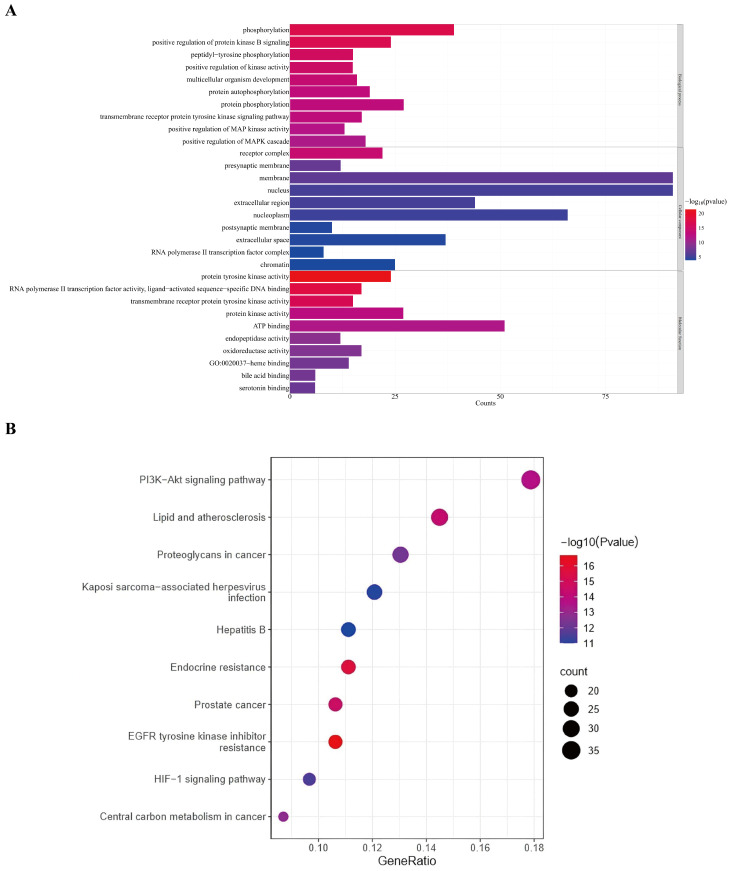
GO and KEGG enrichment analyses for the mechanism of GE in ameliorating alcoholic liver injury. (**A**) GO enrichment analysis indicates the top 10 GO terms in each category at *p* < 0.01. (**B**) KEGG enrichment analysis indicates the top 10 KEGG terms at *p* < 0.01.

To further investigate the mechanisms by which GE contributes to the amelioration of alcoholic liver injury, the Metascape database was employed to conduct GO and KEGG enrichment analyses, with the screening criterion of *p* < 0.01, as shown in Figure 3. The GO enrichment analysis included 196 biological processes (BPs), 28 cellular components (CCs), and 98 molecular functions (MFs). The results indicate that the primary BP items by which GE ameliorates alcoholic liver injury might include phosphorylation, positive regulation of protein kinase B signaling, peptidyl-tyrosine phosphorylation, positive regulation of kinase activity, multicellular organism development, protein autophosphorylation, protein phosphorylation, transmembrane receptor protein tyrosine kinase signaling pathway, positive regulation of MAP kinase activity, and positive regulation of MAPK cascade. The primary CC items might involve receptor complex, presynaptic membrane, membrane, nucleus, extracellular region, nucleoplasm, postsynaptic membrane, extracellular space, RNA polymerase II transcription factor complex, and chromatin. The primary MF items might include protein tyrosine kinase activity, RNA polymerase II transcription factor activity, ligand-activated sequence-specific DNA binding, transmembrane receptor protein tyrosine kinase activity, protein kinase activity, ATP binding, endopeptidase activity, oxidoreductase activity, heme binding, bile acid binding, and serotonin binding. The KEGG pathway enrichment analysis identified 154 signaling pathways. Among them, the primary signaling pathways might involve the PI3K-AKT signaling pathway, lipid and atherosclerosis, proteoglycans in cancer, Kaposi sarcoma-associated herpes virus infection, hepatitis B, endocrine resistance, prostate cancer, EGFR tyrosine kinase inhibitor resistance, HIF-1 signaling pathway, and central carbon metabolism.

### 3.4. Molecular Docking of the Main Bioactive Components Derived from Gastrodia elata to Key Targets

**Figure 4:**
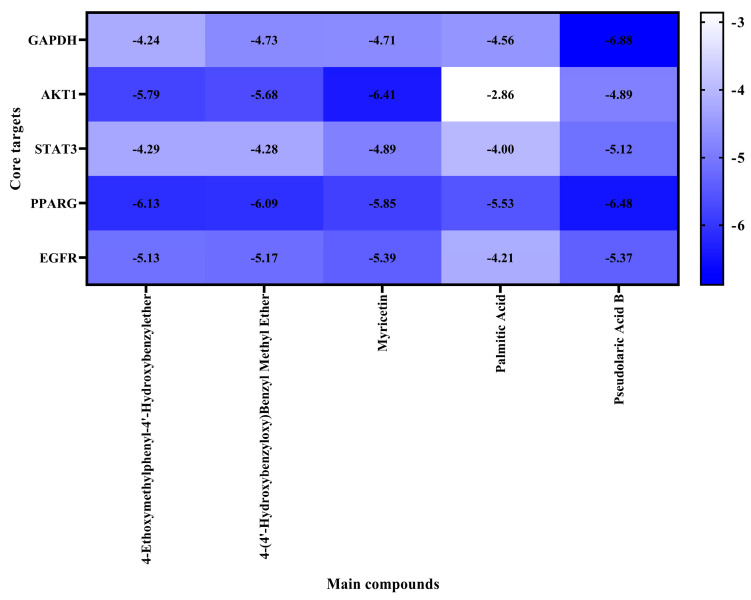
The heatmap of binding energies between the main bioactive components of GE and the key targets associated with alcoholic liver injury. Darker blue rectangles represent lower binding energy scores for docking of ligands to target proteins.

To determine the binding mode of the main bioactive components derived from GE interacting with the key targets, molecular docking simulation was conducted. The results of molecular docking are shown in Figure 4. Normally, a negative binding energy score (less than 0 kcal/mol) indicates that a ligand is likely to bind to its target. A binding energy score more negative than −5.0 kcal/mol typically suggests a strong binding affinity between the ligand and the target. The results of molecular docking suggest that all of the five main components dock well to the five key targets of alcoholic liver injury. Among them, the binding energy scores of pseudolaric acid B to the three key target proteins of GAPDH, PPARG and STAT3 were better than those of the other components, indicating that pseudolaric acid B as the core component of BE might play a starring role in alleviating alcoholic liver injury.

**Figure 5:**
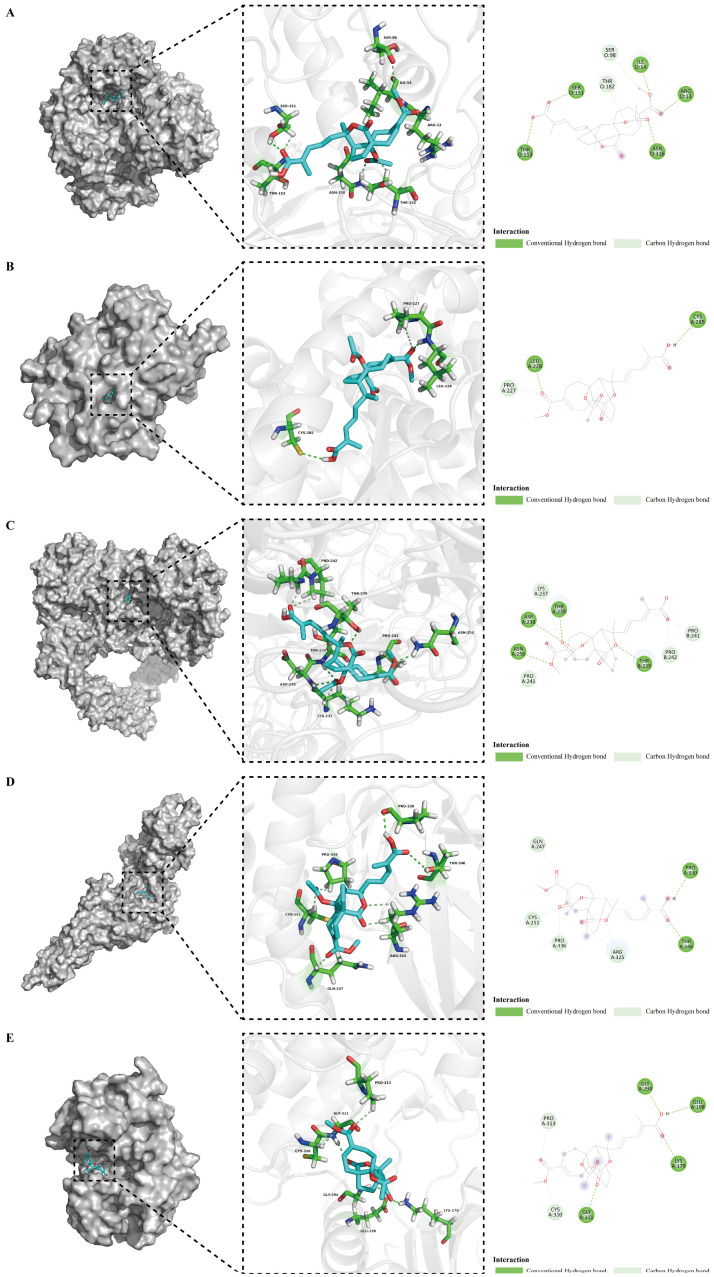
The docking modes between the core bioactive component of GE and the key targets of alcoholic liver injury. (**A**) Binding mode of pseudolaric acid B to GAPDH (PDB ID: 1U8F). (**B**) Binding mode of pseudolaric acid B to PPARG (PDB ID: 6MS7). (**C**) Binding mode of pseudolaric acid B to EGFR (PDB ID: 3NJP). (**D**) Binding mode of pseudolaric acid B to STAT3 (PDB ID: 6NJS). (**E**) Binding mode of pseudolaric acid B to AKT1 (PDB ID: 3MV5).

Subsequently, we utilized Pymol and DiscoveryStudio software to visualize and analyze 3D and 2D models of pseudolaric acid B interaction with the key targets, as shown in Figure 5. Pseudolaric acid B docked to the binding pocket of the five key target proteins. Furthermore, in these docking models, pseudolaric acid B formed hydrogen bonds with several key residues in the target proteins. Specifically, in GAPDH, it interacted with ARG-13, ILE-14, SER-98, SER-151, THR-153, THR-182, and ASN-316. In PPARG, the interacting residues were PRO-227, LEU-228, and CYS-285. In EGFR, the key residues included LYS-237, ASP-238, THR-239, PRO-241, PRO-242, and ASN-256. In STAT3, it formed bonds with GLN-247, CYS-251, ARG-325, PRO-330, PRO-336, and THR-346. In AKT1, the residues involved were LYS-179, GLU-198, GLY-294, CYS-310, GLY-311, and PRO-313. These results suggest that pseudolaric acid B could interact with these key targets of alcoholic liver injury, further verifying the accuracy of our above results.

### 3.5. Verification of Binding of Pseudolaric Acid B to the Key Target Proteins by Using Molecular Dynamics Simulation

**Figure 6:**
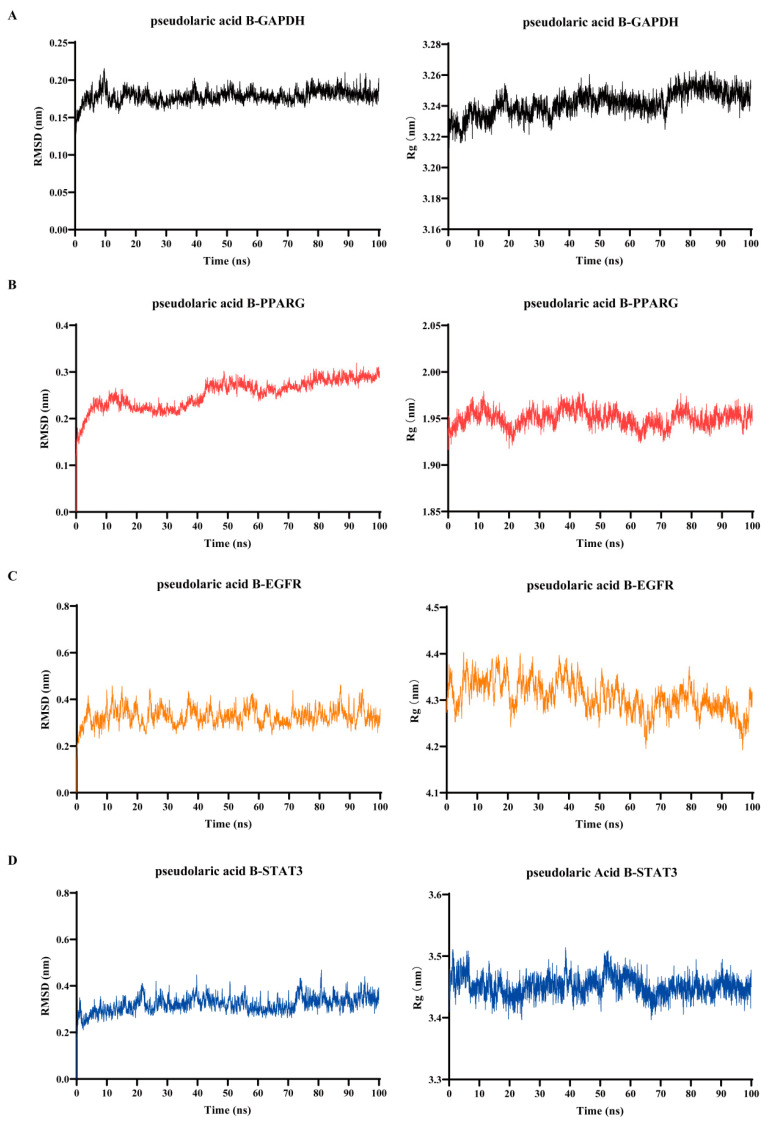
Molecular dynamics simulations of pseudolaric acid B interacted with the key target proteins for 100 ns. (**A**) The RMSD and Rg plots for the complex of pseudolaric acid B interacted with GAPDH. (**B**) The RMSD and Rg plots for the complex of pseudolaric acid B interacted with PPARG. (**C**) The RMSD and Rg plots for the complex of pseudolaric acid B interacted with EGFR. (**D**) The RMSD and Rg plots for the complex of pseudolaric acid B interacted with STAT3.

To evaluate the stability of the predicted binding modes of pseudolaric acid B with its key target proteins, we selected the four optimal binding modes (with binding energy scores more negative than −5.0 kcal/mol) for further molecular dynamics simulations, which were conducted over a 100-ns timescale. As depicted in Figure 6, the root-mean-square deviation (RMSD) plots revealed that the complexes formed by pseudolaric acid B interacting with the key target proteins exhibited minimal stability fluctuations, with deviations less than 0.2 nm. Specifically, the RMSD values for the complexes of pseudolaric acid B-GAPDH, pseudolaric acid B-PPARG, pseudolaric acid B-EGFR, and pseudolaric acid B-STAT3 were 0.178 ± 0.010 nm, 0.253 ± 0.031 nm, 0.328 ± 0.039 nm, and 0.321 ± 0.035 nm, respectively. Additionally, the radius of gyration (Rg) plots indicate that these complexes adopted compact conformations, with Rg values of 3.218 ± 0.258 nm for the pseudolaric acid B-GAPDH complex, 1.937 ± 0.143 nm for the pseudolaric acid B-PPARG complex, 4.304 ± 0.123 nm for the pseudolaric acid B-EGFR complex, and 3.451 ± 0.017 nm for the pseudolaric acid B-STAT3 complex. These results demonstrate that the target proteins maintained stable structures after binding with pseudolaric acid B, thereby validating the credibility of the predicted binding models.

### 3.6. The Effect of Pseudolaric Acid B in Ameliorating Alcoholic Liver Injury via the Regulation of Key Targets

**Figure 7:**
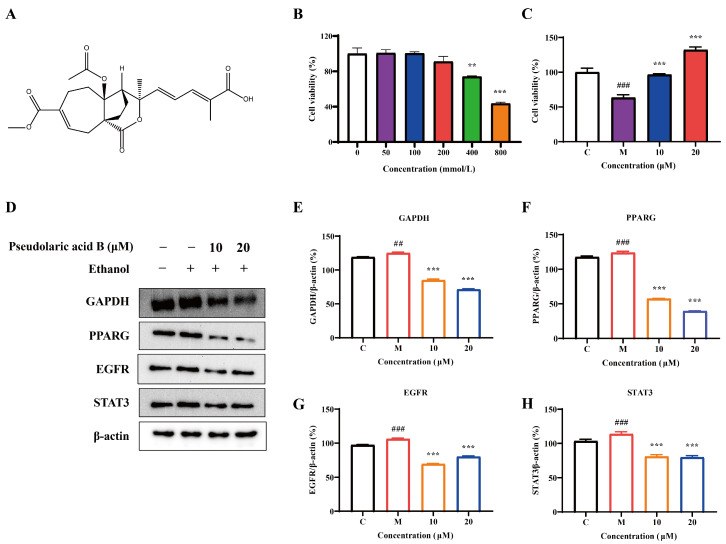
The effect of pseudolaric acid B on the amelioration of alcoholic liver injury in HepG2 cells. (**A**) The chemical structure of pseudolaric acid B. (**B**) The effect of different concentrations of ethanol on HepG2 cell viability. (**C**) Effect of different concentrations of pseudolaric acid B on cell viability in ethanol-induced HepG2 cell injury. (**D**) Western blot assay, conducted to detect the effect of pseudolaric acid B on the expression of the key target proteins. (**E**–**H**) Quantification of the expression levels of GAPDH, PPARG, EGFR, and STAT3. All data represent the average of three independent experiments (mean ± SEM). ## *p* < 0.01, ### *p* < 0.001 vs. the control; ** *p* < 0.01, *** *p* < 0.001 vs. the model.

Given that the above results are based on computational simulation, experiment validation is necessary to confirm these findings. The chemical structure of pseudolaric acid B is shown as Figure 7A. To test the optimal ethanol concentration for inducing alcoholic liver injury in HepG2 cells, various concentrations of ethanol (0, 50, 100, 200, 400, and 800 mmol/L) were designed with a stimulation duration of 24 h. As shown in Figure 7B, when the ethanol concentration reached 400 mmol/L, a significant decline in cell viability was observed. Subsequently, we selected 400 mmol/L of ethanol to induce HepG2 cells and then test the effect of pseudolaric acid B against alcoholic liver injury. The results show that 10 μM and 20 μM of pseudolaric acid B significantly alleviated ethanol-induced HepG2 cell injury (Figure 7C). To further confirm this effect and determine whether it is associated with the key targets, we conducted a Western blot assay to detect the effect of pseudolaric acid B on the expression levels of GAPDH, PPARG, EGFR, and STAT3. As shown in Figure 7D–H, the protein expression levels of GAPDH, PPARG, EGFR, and STAT3 were suppressed by 10 μM and 20 μM of pseudolaric acid B.

**Figure 8:**
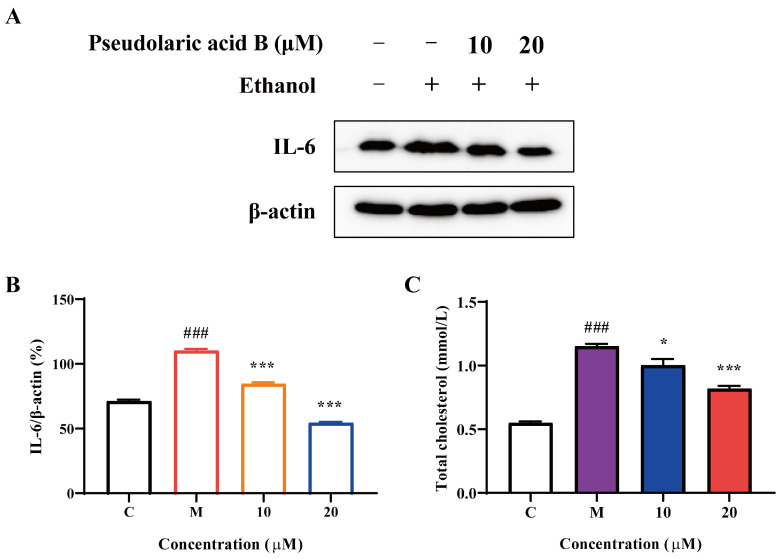
Pseudolaric acid B suppressed the expression of IL-6 and reduced the content of TC in HepG2 cells. (**A**) The expression of IL-6 was detected by Western blot. (**B**) Quantification of the expression levels of IL-6. (**C**) Pseudolaric acid B reduced the content of TC in HepG2 cells. All data represent the average of three independent experiments (mean ± SEM). ### *p* < 0.001 vs. the control; * *p* < 0.05, *** *p* < 0.001 vs. the model.

Alcoholic liver injury induces the release of a substantial amount of inflammatory cytokines from hepatic cells. To further verify the preventive effect of pseudolaric acid B on alcoholic liver injury, we assessed its impact on downstream inflammatory cytokines. As shown in Figure 8A,B, after treatment with 10 μM and 20 μM of pseudolaric acid B, the expression of IL-6 was significantly suppressed compared with the model group. Moreover, the levels of total cholesterol (TC) are correlated with the degree of liver injury. As liver injury progresses, the capacity of liver to synthesize and metabolize cholesterol is further impaired, potentially leading to an increase in TC levels. As shown in Figure 8C, pseudolaric acid B significantly reduced the TC content in HepG2 cells, thereby maintaining normal lipid metabolism. In conjunction with our previous findings, these results further demonstrate that pseudolaric acid B, as a core component of GE, exhibits preventive effects against alcoholic liver injury.

## 4. Discussion

GE is a common medicinal plant in China, widely known for its therapeutic effects of dispelling wind, calming the liver, and relieving wind symptoms, and it plays an important role in the protection of the liver [18,19]. Alcoholic liver injury is a chronic liver condition caused by long-term excessive alcohol consumption, encompassing pathological changes such as alcoholic steatohepatitis, liver cirrhosis, and hepatic fibrosis. In severe cases, it can progress to liver cancer, acute liver failure, and even death [20]. Currently, there is limited research on the use of GE as an adjuvant treatment for alcoholic liver injury. In this study, we aimed to construct an active ingredient-target network for GE in ameliorating alcoholic liver injury using network pharmacology, identify core bioactive ingredients and targets through molecular docking simulation and molecular dynamics simulation, and lay the groundwork for further in vivo studies based on the results of cell experiment validation.

Based on the constructed active ingredient-target network of GE in ameliorating alcoholic liver injury, the top five key active ingredients against alcoholic liver injury are 4-ethoxytoluyl-4′-hydroxyl benzyl methyl ether, 4-hydroxybenzyl methyl ether, pseudolaric acid B, palmitic acid, and myricetin. After PPI network analysis, the top five core targets for GE in alleviating alcoholic liver injury were identified as GAPDH, AKT1, STAT3, PPARG, and EGFR. These key targets play important roles in the treatment of liver diseases. GO enrichment analysis revealed that protein phosphorylation, positive regulation of protein kinase B signaling, and the MAPK kinase signaling pathway are the main enriched pathways for the target genes of GE against alcoholic liver injury. Cellular inflammation leads to oxidative stress and mitochondrial dysfunction, ultimately causing hepatocyte injury, which may further progress alcoholic liver injury to fatty liver [21]. It has been reported that inhibition of phosphorylated-STAT3 reduced inflammation and oxidative stress damage in the livers of mice with alcoholic liver injury [22]. However, activating the positive regulation of protein kinase B signaling and the MAPK kinase pathway produce inflammatory factors and induce various inflammatory diseases [23]. KEGG pathway enrichment analysis showed that the main pathways are concentrated in the PI3K-AKT signaling pathway and lipid metabolism and atherosclerosis. After being activated by PI3K, AKT promotes the proliferation of hepatocellular carcinoma cells and inhibits apoptosis [24]. Atherosclerosis leads to vascular metabolic dysfunction, lipid metabolism disorders, and chronic inflammation, which in turn promotes liver injury [25].

Furthermore, the results of molecular docking indicate that the five main bioactive components of GE identified in this study stably bound to key targets such as GAPDH, AKT1, STAT3, PPARG, and EGFR, with relatively low binding energy scores. Among them, pseudolaric acid B possessed the strongest binding ability to most of the key target proteins compared to other components, which may highlight its crucial role in the efficacy of GE against alcoholic liver injury. In these binding models, pseudolaric acid B appears to bind to the sites of NAD+ (N316) and D-glyceraldehyde 3-phosphate (S151-T153), suggesting that it may act as a competitive inhibitor in ameliorating liver alcoholic injury. Additionally, pseudolaric acid B appears to bind to domain II of the EGFR extracellular domain, which is a key domain in dimerization of EGFR. The dimerization of the EGFR extracellular domain triggers the activation of the EGFR kinase domain, resulting in the transduction of EGFR and its downstream pathway, such as AKT and STAT3 [26]. In our previous study, caffeine and gallic acid were also found to alleviate liver disease via the EGFR extracellular domain [16,27]. The subsequent results of molecular dynamics simulations further validated the stability and reliability of the binding models of pseudolaric acid B to these key target proteins. These results highlight the importance of pseudolaric acid B as a core component of BE in alleviating alcoholic liver injury.

In this study, to further validate the results of network pharmacology and molecular simulations, we carried out cell experiments, including MTT assay and Western blotting. The results showed that pseudolaric acid B significantly ameliorated ethanol-induced HepG2 injury via down-regulating the protein expression of the key targets, including GAPDH, PPARG, EGFR, and STAT3. Additionally, the agent also suppressed IL-6. Among these targets, AKT and STAT3 are key proteins in the downstream pathway of EGFR [26,28]. Activation of EGFR triggers abnormal activation of AKT and STAT3, resulting in a cascade of downstream signaling molecule reactions. Abnormal activation of AKT may interfere with normal cellular metabolism and regulatory mechanisms, leading to metabolic disorders in hepatocytes and worsening liver injury [29]. Similarly, abnormal activation of STAT3 may cause excessive release of inflammatory factors in the liver, resulting in further damage to liver tissue [30]. The reduction of inflammatory factors will further weaken the induction of PPARG and GAPDH protein expression by inflammatory signals, thereby indirectly reducing the expression of PPARG and GAPDH. As a key enzyme in glycolysis, the reduced expression of GAPDH may affect the energy metabolism process of hepatocytes [31]. IL-6 is commonly used as a downstream inflammatory factor in the liver to verify the anti-inflammatory effects of drugs [32]. Moreover, the activation of the EGFR pathway induces the upregulation of IL-6 through STAT3, causing damage to hepatocytes [33]. Therefore, pseudolaric acid B may regulate the downstream pathway of EGFR to reduce the production of inflammatory factors, thereby suppressing the expression of related proteins. To further verify the preventive effect of pseudolaric acid B against alcoholic liver injury, we measured the TC content in HepG2 cells. The results show that pseudolaric acid B maintained normal lipid metabolism in the liver by reducing TC levels in liver cells, thereby alleviating ethanol-induced TC accumulation. While HepG2 cells may not fully capture the complexity of alcoholic liver injury observed in humans or animal models, our findings offer a valuable scientific basis for the prevention and treatment of alcoholic liver disease. In the future studies, more experiments, including in vivo assays, are needed to further validate these findings.

## 5. Conclusions

In summary, our study demonstrates the potential targets and bioactive components of GE through constructing an interaction network architecture between GE and alcoholic liver injury using network pharmacology. Further studies of molecular docking, molecular dynamics simulation, and in vitro assay verified the efficacy and molecular mechanism of pseudolaric acid B, the core bioactive component derived from GE, in ameliorating alcoholic liver injury. Our findings will provide a scientific basis for the precise development and utilization of GE in functional foods.

## Figures and Tables

**Table 1:** The active components derived from *Gastrodia elata*.

No.	Ingredient ID	Component Name	Formula
1	TCMC4620	4-(4′-Hydroxybenzyloxy)benzyl methyl ether	C_13_H_12_O_2_
2	TCMC4547	4-Ethoxymethylphenyl-4′-hydroxybenzylether	C_16_H_18_O_3_
3	TCMC478	4-[(4-Hydroxyphenyl)methyl]phenol	C_19_H_20_O_2_
4	TCMC495	4-Hydroxybenzaldehyde	C_8_H_6_O_3_
5	TCMC2829	4-Hydroxybenzyl alcohol	C_7_H_8_O_2_
6	TCMC3279	4-Hydroxybenzyl methyl ether	C_8_H_10_O_2_
7	TCMC497	4-Hydroxybenzylamine	C_7_H_9_NO
8	TCMC631	Adenine	C_5_H_5_N_5_
9	TCMC632	Adenosine	C_10_H_13_N_5_O_4_
10	TCMC774	Benzyl alcohol	C_7_H_8_O
11	TCMC801	Beta-sitosterol	C_29_H_50_O
12	TCMC2223	Vanillin	C_8_H_8_O_3_
13	TCMC4448	Bis(4-hydroxybenzyl)ether	C_14_H_14_O_3_
14	TCMC923	Citric acid	C_6_H_8_O_7_
15	TCMC995	Daucosterol	C_35_H_60_O_6_
16	TCMC1072	Disogenin	C_27_H_42_O_4_
17	TCMC4578	Gastrodamine	C_14_H_15_NO_3_
18	TCMC3409	Gastrodin	C_13_H_18_O_7_
19	TCMC1298	Guanosine	C_10_H_13_N_5_O_5_
20	TCMC1609	Myricetin	C_15_H_10_O_8_
21	TCMC1830	Palmitic acid	C_16_H_32_O_2_
22	TCMC1839	Paraben	C_7_H_6_O_3_
23	TCMC3338	P-Hydroxybenzyl ethyl ether	C_9_H_12_O_2_
24	TCMC1955	Protocatechualdehyde	C_7_H_6_O_3_
25	TCMC2821	Pseudolaric acid B	C_23_H_28_O_8_
26	TCMC5840	Bis(4-hydroxybenzyl)ether mono-β-D-glucopyranoside	C_6_H_12_O_6_
27	TCMC2111	Succinic acid	C_4_H_6_O_2_
28	TCMC2886	Sucrose	C_12_H_22_O_11_
29	TCMC5445	Tris-[4-(β-D-glucopyranosyloxy)benzyl]citrate	C_45_H_56_O_25_
30	TCMC2209	Uridine	C_9_H_12_N_2_O_6_
31	TCMC2222	Vanillic acid	C_8_H_8_O_4_

## Data Availability

The original contributions presented in this study are included in the article. Further inquiries can be directed to the corresponding authors.

## Abbreviations

The following abbreviations are used in this manuscript:

GE	*Gastrodia elata*
GO	Gene ontology
KEGG	Kyoto Encyclopedia of Genes and Genomes
ALD	Alcoholic liver disease
MD	Molecular dynamics
MTT	Methyl thiazolyl tetrazolium
SDS-PAGE	Sodium dodecyl sulfate–polyacrylamide gel electrophoresis
PPI	Protein–protein interaction
DMSO	Dimethyl sulfoxide
RMSD	Root-mean-square deviation
Rg	Radius of gyration

## References

[1] Lai Y., Tan Q., Xv S., Huang S., Wang Y., Li Y., Zeng T., Mo C., Chen Y., Huang S. (2021). Ginsenoside Rb1 Alleviates Alcohol-Induced Liver Injury by Inhibiting Steatosis, Oxidative Stress, and Inflammation. Front. Pharmacol..

[2] Wang W.J., Xiao P., Xu H.Q., Niu J.Q., Gao Y.H. (2019). Growing burden of alcoholic liver disease in China: A review. World J. Gastroenterol..

[3] Li Y., Wang S., Ni H.M., Huang H., Ding W.X. (2014). Autophagy in alcohol-induced multiorgan injury: Mechanisms and potential therapeutic targets. Biomed Res. Int..

[4] Dukić M., Radonjić T., Jovanović I., Zdravković M., Todorović Z., Kraišnik N., Aranđelović B., Mandić O., Popadić V., Nikolić N. (2023). Alcohol, Inflammation, and Microbiota in Alcoholic Liver Disease. Int. J. Mol. Sci..

[5] Singal A.K., Shah V.H. (2019). Current trials and novel therapeutic targets for alcoholic hepatitis. J. Hepatol.

[6] Zhang S., Yang Y., Zhang R., Gao J., Wu M., Wang J., Sheng J., Sun P. (2024). The Potential Mechanism of Alpiniae oxyphyllae Fructus Against Hyperuricemia: An Integration of Network Pharmacology, Molecular Docking, Molecular Dynamics Simulation, and In Vitro Experiments. Nutrients.

[7] Bildziukevich U., Özdemir Z., Wimmer Z. (2019). Recent Achievements in Medicinal and Supramolecular Chemistry of Betulinic Acid and Its Derivatives. Molecules.

[8] Yi Z., Yue Y., Kan J., Wang Z., Awad S., Ibrahim A., Du M. (2024). Biocontrol of Fusarium oxysporum-infested Gastrodia elata Bl. by *Lactobacillus curvatus* 2768-VOCs and mechanism of inhibition. Food Biosci..

[9] Ahmad O., Wang B., Ma K., Deng Y., Li M., Yang L., Yang Y., Zhao J., Cheng L., Zhou Q. (2019). Lipid Modulating Anti-oxidant Stress Activity of Gastrodin on Nonalcoholic Fatty Liver Disease Larval Zebrafish Model. Int. J. Mol. Sci..

[10] Qu L.L., Yu B., Li Z., Jiang W.X., Jiang J.D., Kong W.J. (2016). Gastrodin Ameliorates Oxidative Stress and Proinflammatory Response in Nonalcoholic Fatty Liver Disease through the AMPK/Nrf2 Pathway. Phytother. Res..

[11] Tuo Y., Lu X., Tao F., Tukhvatshin M., Xiang F., Wang X., Shi Y., Lin J., Hu Y. (2024). The Potential Mechanisms of Catechins in Tea for Anti-Hypertension: An Integration of Network Pharmacology, Molecular Docking, and Molecular Dynamics Simulation. Foods.

[12] Challapa-Mamani M.R., Tomás-Alvarado E., Espinoza-Baigorria A., León-Figueroa D.A., Sah R., Rodriguez-Morales A.J., Barboza J.J. (2023). Molecular Docking and Molecular Dynamics Simulations in Related to *Leishmania donovani*: An Update and Literature Review. Trop. Med. Infect. Dis..

[13] Wang J., Xian J., Zhang R., Wang Z., Zhang S., Zhao D., Sheng J., Sun P. (2025). α-Mangostin Exhibits Antitumor Activity Against NCI-H1975 Cells via the EGFR/STAT3 Pathway: An Experimental and Molecular Simulation Study. Molecules.

[14] Hildebrand P.W., Rose A.S., Tiemann J.K.S. (2019). Bringing Molecular Dynamics Simulation Data into View. Trends. Biochem. Sci..

[15] Hou W., Wei B., Liu H.S. (2021). The Protective Effect of Panax notoginseng Mixture on Hepatic Ischemia/Reperfusion Injury in Mice via Regulating NR3C2, SRC, and GAPDH. Front. Pharmacol..

[16] Zhang D., Zhou Q., Yang X., Zhang Z., Wang D., Hu D., Huang Y., Sheng J., Wang X. (2024). Gallic Acid Can Promote Low-Density Lipoprotein Uptake in HepG2 Cells via Increasing Low-Density Lipoprotein Receptor Accumulation. Molecules.

[17] Wei J., Zhao X., Long F., Tian K., Wu L. (2024). Lianhua Qingwen exerts anti-liver cancer effects and synergistic efficacy with sorafenib through PI3K/AKT pathway: Integrating network pharmacology, molecular docking, and experimental validation. Gene.

[18] Wu Y.N., Wen S.H., Zhang W., Yu S.S., Yang K., Liu D., Zhao C.B., Sun J. (2023). Gastrodia elata BI: A Comprehensive Review of Its Traditional Use, Botany, Phytochemistry, Pharmacology, and Pharmacokinetics. Evid-Based. Compl. Alt..

[19] Seok P.R., Kim J.H., Kwon H.R., Heo J.S., Choi J.R., Shin J.H. (2018). Protective effects of Gastrodia elata Blume on acetaminophen-induced liver and kidney toxicity in rats. Food Sci. Biotechnol..

[20] Zhang Y., Zhang Y., Zhou S., Rehman M.U., Lin F., Zhang J., Zhou H. (2025). HTR1D regulates the PI3K/Akt signaling pathway to impact hepatocellular carcinoma development and resistance to sorafenib. BMC Cancer.

[21] Park W.Y., Song G., Noh J.H., Kim T., Kim J.J., Hong S., Park J., Um J.Y. (2021). Raphani Semen (*Raphanus sativus* L.) Ameliorates Alcoholic Fatty Liver Disease by Regulating De Novo Lipogenesis. Nutrients.

[22] Tan Y., Zhang F., Fan X., Lu S., Liu Y., Wu Z., Huang Z., Wu C., Cheng G., Li B. (2023). Exploring the effect of Yinzhihuang granules on alcoholic liver disease based on pharmacodynamics, network pharmacology and molecular docking. Chin. Med..

[23] An L., Zhao J., Sun X., Zhou Y., Zhao Z. (2020). S-allylmercaptocysteine inhibits mucin overexpression and inflammation via MAPKs and PI3K-Akt signaling pathways in acute respiratory distress syndrome. Pharmacol. Res..

[24] Zhang Z., Xu C.M., Chen W., Yao K.T., Sun T., Wang J.H. (2025). Global, regional, and national burdens of alcohol-related cirrhosis among women from 1992 to 2021 and its predictions. Sci. Rep..

[25] Stefanadis C., Antoniou C.K., Tsiachris D., Pietri P. (2017). Coronary Atherosclerotic Vulnerable Plaque: Current Perspectives. J. Am. Heart. Assoc..

[26] Sun P., Zhang S., Qu Y., Li X., Chen G., Wang X., Sheng J., Wang J. (2024). Coccinic acid exhibits anti-tumor efficacy against NSCLC harboring EGFR L858R/T790M mutation via the EGFR/STAT3 pathway. Bioorg. Chem..

[27] Huang Y.W., Wang L.T., Zhang M., Nie Y., Yang J.B., Meng W.L., Wang X.J., Sheng J. (2023). Caffeine can alleviate non-alcoholic fatty liver disease by augmenting LDLR expression via targeting EGFR. Food Funct..

[28] Wang J., Wang Y., Zhang S., Qu Y., Zhang R., Wang X., Sheng J., Sun P. (2024). Inhibitory effect of 1,4,5,6-tetrahydroxy-7,8-diprenylxanthone against NSCLC with L858R/T790M/C797S mutant EGFR. Sci. Rep..

[29] Reyes-Gordillo K., Shah R., Arellanes-Robledo J., Cheng Y., Ibrahim J., Tuma P.L. (2019). Akt1 and Akt2 Isoforms Play Distinct Roles in Regulating the Development of Inflammation and Fibrosis Associated with Alcoholic Liver Disease. Cells.

[30] Horiguchi N., Wang L., Mukhopadhyay P., Park O., Jeong W.I., Lafdil F., Osei-Hyiaman D., Moh A., Fu X.Y., Pacher P. (2008). Cell type-dependent pro- and anti-inflammatory role of signal transducer and activator of transcription 3 in alcoholic liver injury. Gastroenterology.

[31] Adamus G. (2017). Impact of Autoantibodies against Glycolytic Enzymes on Pathogenicity of Autoimmune Retinopathy and Other Autoimmune Disorders. Front. Immunol..

[32] Zhang J.X., Yang Y., Huang H., Xie H.B., Huang M., Jiang W., Ding B.W., Zhu Q.X. (2022). TNF-α/TNFR1 regulates the polarization of Kupffer cells to mediate trichloroethylene-induced liver injury. Ecotox. Environ. Safe..

[33] Yu H., Lee H., Herrmann A., Buettner R., Jove R. (2014). Revisiting STAT3 signalling in cancer: New and unexpected biological functions. Nat. Rev. Cancer.

